# Classifying Incomplete Gene-Expression Data: Ensemble Learning with Non-Pre-Imputation Feature Filtering and Best-First Search Technique

**DOI:** 10.3390/ijms19113398

**Published:** 2018-10-30

**Authors:** Yuanting Yan, Tao Dai, Meili Yang, Xiuquan Du, Yiwen Zhang, Yanping Zhang

**Affiliations:** 1 School of Computer Science and Technology, Anhui University, Hefei 230601, China; ytyan2016@163.com (Y.Y.); vc1024@163.com (T.D.); yangml1995y@163.com (M.Y.); zhangyiwen@ahu.edu.cn (Y.Z.); 2 Key Laboratory of Intelligent Computing and Signal Processing of Ministry of Education, Anhui University, Hefei 230601, China

**Keywords:** gene-expression data, feature selection, best first search, classification

## Abstract

(1) Background: Gene-expression data usually contain missing values (MVs). Numerous methods focused on how to estimate MVs have been proposed in the past few years. Recent studies show that those imputation algorithms made little difference in classification. Thus, some scholars believe that how to select the informative genes for downstream classification is more important than how to impute MVs. However, most feature-selection (FS) algorithms need beforehand imputation, and the impact of beforehand MV imputation on downstream FS performance is seldom considered. (2) Method: A modified chi-square test-based FS is introduced for gene-expression data. To deal with the challenge of a small sample size of gene-expression data, a heuristic method called recursive element aggregation is proposed in this study. Our approach can directly handle incomplete data without any imputation methods or missing-data assumptions. The most informative genes can be selected through a threshold. After that, the best-first search strategy is utilized to find optimal feature subsets for classification. (3) Results: We compare our method with several FS algorithms. Evaluation is performed on twelve original incomplete cancer gene-expression datasets. We demonstrate that MV imputation on an incomplete dataset impacts subsequent FS in terms of classification tasks. Through directly conducting FS on incomplete data, our method can avoid potential disturbances on subsequent FS procedures caused by MV imputation. An experiment on small, round blue cell tumor (SRBCT) dataset showed that our method found additional genes besides many common genes with the two compared existing methods.

## 1. Introduction

As an important technology in the field of bioinformatics, microarray technology is prominent do to its ability to potentially simultaneously measure thousands of gene-expression levels [1,2]. Gene-expression data obtained from microarray experiments are usually confronted with high-dimension and missing-data problems [3,4]. This characteristic generates two problems for downstream gene-expression data analysis (e.g., classification). The first is that data obtained from microarray technology often contains missing values (MVs). MVs present a challenge to traditional analysis models that require a complete data matrix [5,6]. Another problem is the high computational complexity caused by data’s high dimensionality [7,8].

Numerous methods were proposed to solve the above two problems in the past few years. MV imputation is the mainstream method that replaces lost entries by an estimation value or a given fixed value [9,10,11,12,13]. Typical representative methods include least-square adaptive (LSA) [9], local least squares (LLS) [10], Bayesian principal component analysis (BPCA) [11], k nearest neighbor (KNN) [12], and partial least square (PLS) [13]. Most MV imputation algorithms applied root mean squared error (RMSE) or its variants as evaluation criteria for the performance of MV imputation algorithms.

In the last decade, several papers focused on the comparison of the impact of different MV imputation methods for downstream statistical analyses [2,14,15,16,17,18,19,20,21]. Those papers often perform a number of typical methods on several datasets to evaluate the biological impact of downstream analyses, such as biomarker detection and gene-clustering analysis or classification. In Reference [2], the authors found that, among those three statistical analyses, classification was the least sensitive to the choice of imputation method. Other papers also found that some imputation algorithms may be competitive with regard to the selected comparison datasets, but no algorithm is uniformly superior in all datasets [15,16,17,18,19,20]. In Reference [21], the authors applied several MV imputation algorithms on real incomplete cancer gene-expression data to evaluate the performance of each method, and they drew the same conclusion: imputation methods have minor impact on classification.

With respect to the computational complexity caused by the high dimension of gene-expression data, FS is the most common and effective technique to deal with this challenge. Feature selection (FS) aims at selecting informative genes from thousands of genes and uncovers the most relevant biological variables. Many FS algorithms have been proposed in the past few years [22,23,24,25,26,27,28,29,30], and they can generally be roughly divided into four groups [31]: filter, wrapper, embedded, and ensemble strategies. As indicated in Reference [31], many algorithms share common elements, merely differing on details from each other among various methods. Moreover, most FS algorithms for gene-expression data require a complete data matrix as the input, but FS algorithms designed for incomplete data are rare.

As gene-expression data often contain MVs, common practice is to apply MV imputation before conducting FS. One may worry whether MV imputation would affect the FS result. For example, in the preprocess phase, features with more than 50% missing data are often directly discarded. It is worth noting that a feature (gene) may still have a greater degree of relevance (discriminative power) than some complete features although it has more than 50% MVs. Simply removing those incomplete features based on missing rates may be inappropriate in some cases. MV imputation methods usually also need to rely on assumptions about data distribution or missing mechanisms, such as the missing-at-random (MAR) assumption [32]. Unfortunately, no technique exists to verify the MAR assumption [33]. Thus, one problem that needs to be considered is whether downstream FS would be influenced when the assumption is violated.

Our motivation was to introduce a FS algorithm that can handle incomplete gene-expression data without previous MV imputation. Thus, it could avoid the potential impact of MV imputation on subsequent FS results. After FS, we applied MV imputation to the selected feature subset to generate a complete data matrix that could be processed by a traditional machine-learning technique, and the best-first search strategy was applied to search the best top features on the selected data subset. We first introduced a modified chi-square test-based FS technique to select informative features from incomplete data. To meet the challenge of the small sample size characteristic of cancer gene-expression data, a heuristic method called recursive element aggregation was designed. After that, a wrapper methodology and forward best-first search strategy were utilized to identify the best top percentage of genes on the selected feature subset. Moreover, biological inference was conducted on the small, round blue cell tumor (SRBCT) dataset to validate the effectiveness of our method.

## 2. Results

### 2.1. Feature-Selection Threshold for MCFS

The MCFS threshold was uniformly evaluated through an experiment in this study. To determine the value of α, the parameter was gradually decreased from 0.01 to 0.00001. For each value of α, we first imputed the MVs on the selected feature subset with the mean value of the observation value of each gene, and then we conducted V-ELM with tenfold cross-validation on the complete feature subset. Finally, the V-ELM performance under each value of α was used to determine the threshold.

Table 1 gives the #.genes under various thresholds. When α = 0.00001, the dataset risinger only had one feature, so α should be bigger than 0.00001. As we can see, with the decrease of α, the #.features were declined. The feature subset selected by MCFS with a smaller α was included in the feature subset selected with bigger α.

Prediction accuracy corresponds to the other five values of α is reported in Table 2. Generally speaking, with α declined from 0.01 to 0.0005, v-elm accuracy is increased gradually. The phenomenon illustrates that more features are removed with the decrease of α, while accuracy increases. It is worth noting that algorithm accuracy increased in most of the datasets (nine of 12), while the performance on garber, liang and risinger declined, especially in liang (about 3% decline) and risinger (about 6% decline). This means that some important genes related to classification were removed when α = 0.0001. Consider the characteristic of MCFS, the feature subset selected by smaller α was included in the feature subset selected by bigger α. The genes selected with α = 0.0005 were also included in the gene subset selected with α = 0.001. Synthesizing the above results, the value of α was reasonable, between [0.0005, 0.001], and we set α = 0.001 in this work.

**Figure 1:**
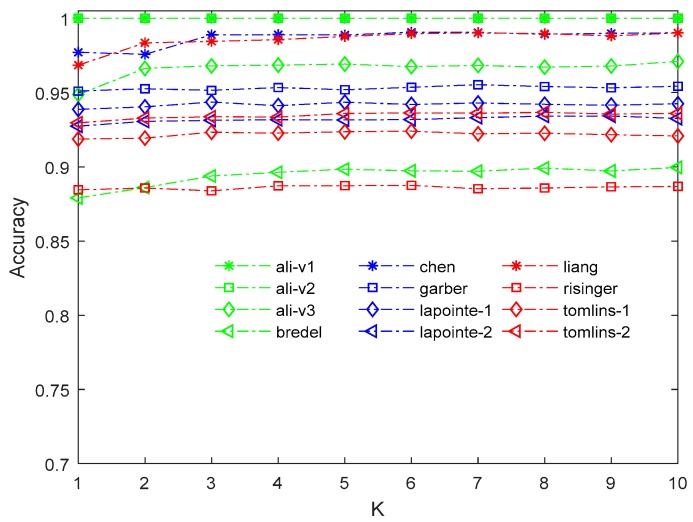
Relationship between FB-MCFS performance and threshold *k* of mean imputation.

Another parameter that needed to be determined was the *k* value of the FBFS method. In FB-MCFS, when algorithm performance does not begin to increase, the next *k* rounds are still evaluated to testify whether the current performance is the best. Figure 1 gives the relationship between *k* performance and value. Similarly, mean value imputation is applied on the feature subset selected by MCFS; then, FB-MCFS is conducted on the complete matrix. 

As depicted in Figure 1, algorithm performance is insensitive with the value of *k* on several datasets (six of 12). For the other six datasets, ali-v3, bredel, chen, liang, lapointe-1, and tomlins-1, when *k* = 3, algorithm performance was nearly unchanged with the increase of *k*. The relationship between the performance and the value of *k* with *KNN* imputation was also conducted. We found the same phenomenon in the experiment. Detailed results of the experiment are in the Supplementary Material. Thus, we suggest to set *k* between [3,6], and we set 5 as the FB-MCFS parameter in this paper.

### 2.2. MCFS with or without Preimputation

To validate the effectiveness of MCFS in avoiding potential disturbance in subsequent classification performance that is caused by MV preimputation, a comparison experiment was conducted. For each algorithm, the average results of 20 trails of tenfold cross-validation experiments were reported in Table 3 (best performance is in bold). The last row gives the average reports on all 12 datasets. MCFS1 represents the method by first conducting MCFS and then conducting MV imputation on the selected feature subset. MCFS2 represents the method by first-conduct MV imputation on the original incomplete gene-expression dataset, and then conducting MCFS on the complete obtained dataset.

As one can see, generally, MCFS1 has better performance than MCFS2, having a 0.69%, 0.51%, and 0.3% average improvement with three imputation methods, respectively. Specifically, the improvement is more than 1% on several datasets (alizadeh-v3, chen, lapointe-2, and tomlins-v2 under BPCA; bredel, tomlins-v1, and tomlins-v2 under KNN; and bredel and tomlins-v1 under MEAN). This indicates that beforehand imputation affects downstream FS in terms of classification. Note: BPCA, Bayesian MV estimation; KNN, k-nearest neighbor imputation; MEAN, mean value imputation.

### 2.3. Comparison of Algorithm Stability under Three Imputation Methods

Recent research shows that MV imputation has minor impact on classification. Here, we aim to study whether MV imputation would affect the following FS and the corresponding classification performance. To this end, we adopted three imputation methods, BPCA [11], KNN [12], and MEAN. For each of the data subsets selected by an FS method with one imputation technique, 20-trail tenfold cross-validation of V-ELM was conducted to give an average result.

Table 4 gives the results (accuracy) of FS algorithms under various MV imputation methods. Performance with more than 0.02 (2%) differences under the three imputation methods is reported in bold. One can see that MCFS is much more stable. In other words, the four comparison FS algorithms are sensitive with MV imputation. The differences in MCFS accuracy are all smaller than 0.02 in all twelve datasets, while NCA, PCA UFF, and ReliefF had six, five, five, and four datasets bigger than 0.02, respectively. It is worth noting that NCA is much more sensitive in the bredel dataset (with a 17 percent difference between KNN and the other two imputation methods). Moreover, UFF was unavailable on the bredel dataset because there were no features selected by UFF. UFF was also sensitive in datasets tom1 and tom2. In general, MCFS is more stable than the FS algorithms compared under the three imputation methods. In other words, this means that beforehand MV imputation would affect the following FS, which needs complete matrix.

### 2.4. Comparison of Gene-Classification Analyses by MCFS and Other Methods

**Figure 2:**
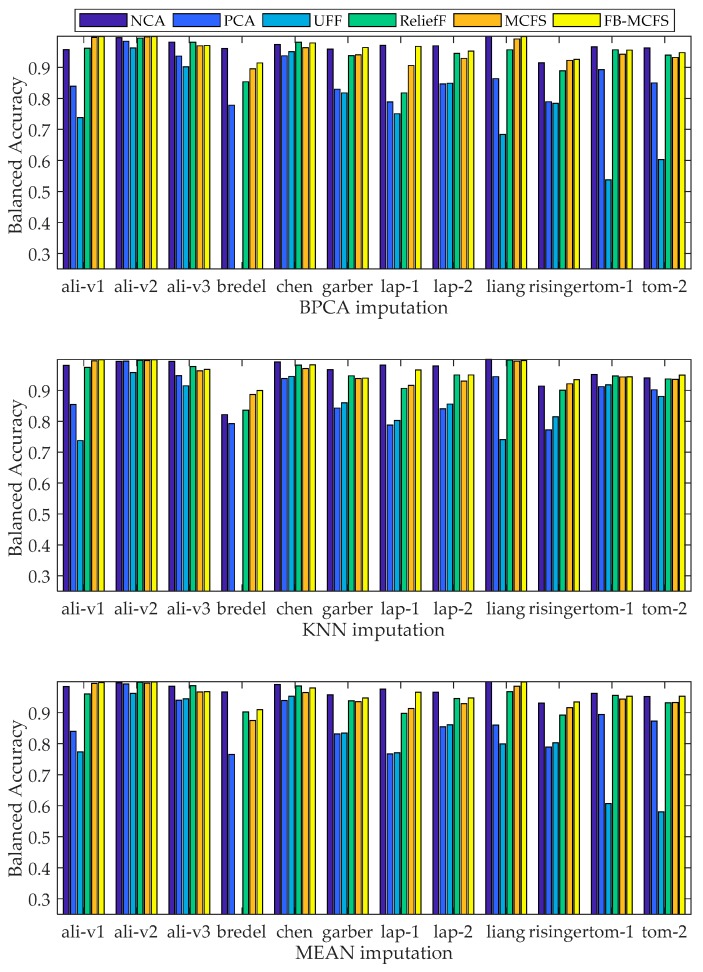
Comparison of balanced accuracies with three imputation methods, respectively.

Classification of FB-MCFS was compared with several algorithms under three different imputation methods, respectively. Our objective was to validate the effectiveness of the best-first search strategy. Thus, for convenience, we sequentially increased the feature subset with a given gene percentage for all the datasets. It is worth to note that, the liang dataset only had 37 samples; when we applied tenfold cross-validation, there were only three test samples, and they sometimes had the same class label, which made AUC calculation impossible. Thus, here we applied fivefold cross-validation, and several evaluation metrics were reported. A one-versus-rest strategy was also applied in this study. Figure 2 gives the results on balanced accuracy. Detailed results of the experiments on evaluation metrics in this study and common genes selected by the FS methods NCA, UFF, ReliefF, and MCFS on dataset alizadeh-v3 appear in the Supplementary Material.

As shown in Figure 2, compared with MCFS, FB-MCFS can improve balanced accuracy on almost all of the datasets. Compared with NCA, PCA, UFF, and ReliefF, FB-MCFS had the best performance in five of 12, five of 12, and four of 12 datasets under three imputation methods, respectively. Because of the unstable performance of the FS algorithms that need beforehand MV imputation, algorithm performance cannot have consistent results. For example, for the bredel dataset, NCA performance was better than FB-MCFS with BPCA and MEAN imputation. However, NCA had a significant gap in balanced accuracy when compared with FB-MCFS under KNN imputation. In general, experiment results demonstrated the effectiveness of FB-MCFS and the stability of MCFS by filtering features without beforehand MV imputation.

Algorithm performance was statistically compared with the Friedman statistical test for multiple comparisons between FB-MCFS and the other five algorithms according to the procedures described in Reference [34]. Table 5 gives the results; *p* values below 0.05 are reported in bold.

### 2.5. Gene and Pathway Analyses for the SRBCT Dataset by MCFS

In this section, study on an SRBCT dataset (according to Reference [35]) is outlined to validate whether the proposed feature-evaluation criterion is biologically meaningful.

#### 2.5.1. Selecting Most Relevant Genes with MCFS

**Figure 3:**
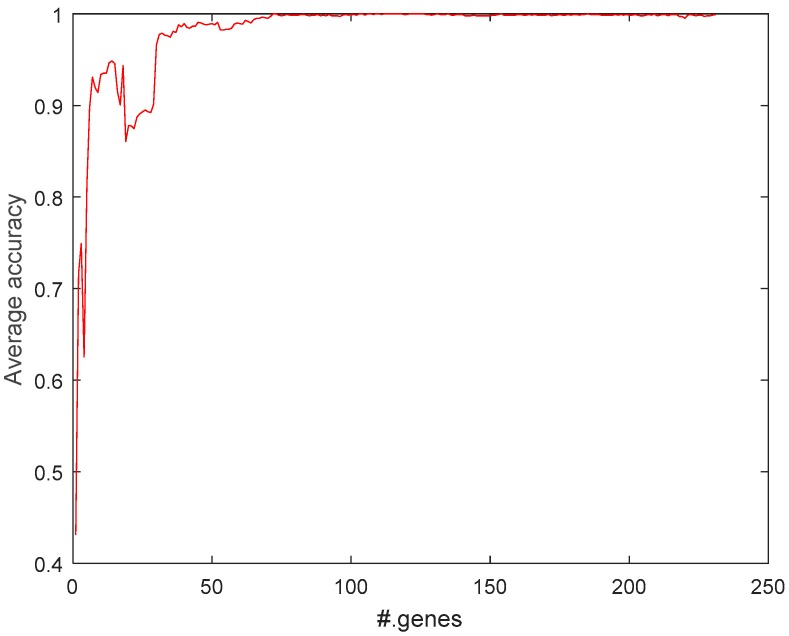
Average accuracy of 100 trials of v-elm trained on selected genes.

**Figure 4:**
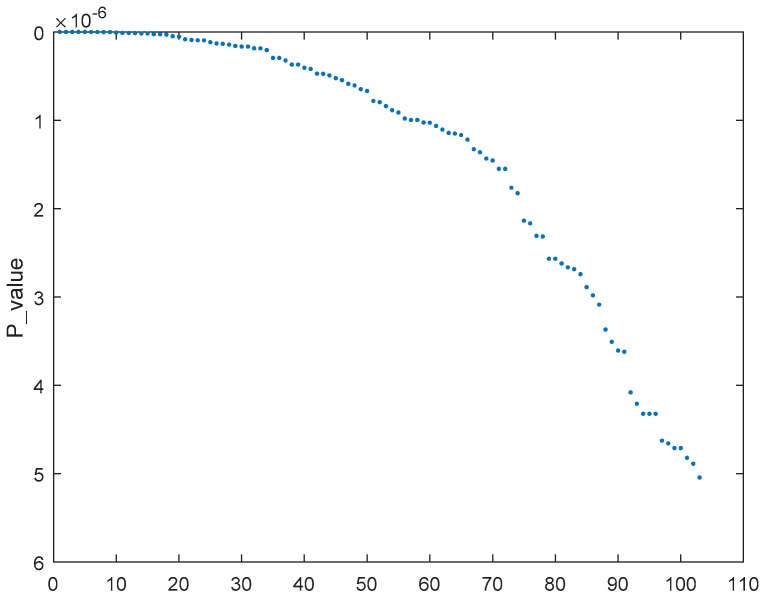
*p*-value corresponding to the 103 selected genes.

To select the most relevant genes in SRBCT, MCFS was used to select 231 genes with a 0.0001 threshold. After that, to get the most accurate selection of relevant genes, genes were ranked based on the *p*-value. Finally, genes were incrementally added to a feature subset and 100 trails of v-elm were conducted to evaluate the feature subset in terms of the average accuracy of the 100 v-elms. Figure 3 reports the top #.genes versus the corresponding average accuracy. 103 genes were selected by our method. Figure 4 reports the *p*-value of the 103 genes. A smaller *p*-value means a greater relevance degree (higher ranking). 

Among the 103 genes, 45 and 27 of the genes were common with the genes selected in References [36,37], respectively. The top 30 genes are given in Table 6. Among the top 30 genes, 18 and 10 genes were reported in References [36,37], respectively. Details about the 103 genes can be seen in the Supplementary Material.

#### 2.5.2. Function Analysis of the Selected Genes

The small, round blue cell tumors (SRBCTs) tend to occur in childhood. SRBCTs include neuroblastoma, non-Hodgkin lymphoma, rhabdomyosarcoma, and the Ewing family of tumors, and they have similar appearance on routine histology. Chromosomal abnormality analysis and molecular probes are usually used to help pathologists.

**Figure 5:**
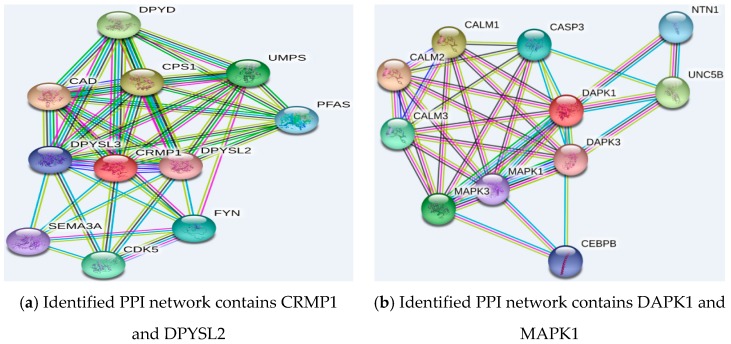

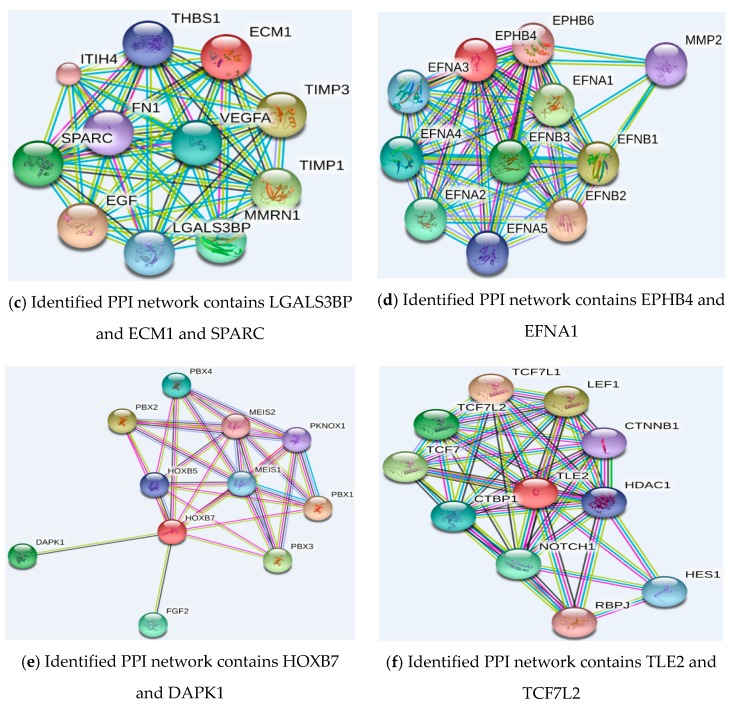
Experimentally identified protein–protein interaction (PPI) network containing the reported genes. Original figures appear in the Supplementary Material.

Several genes from Table 6 corroborate with each other according to existing research results. Figure 5 gives the protein–protein interaction (PPI) networks that were experimentally validated [38]. For classification, some of the listed genes that appeared in PPI network can indicate the importance of the validated biological process in the task. Generally speaking, several genes are involved in Wnt (wingless related integration) signaling (e.g., TLE2 (transducin-like enhancer of split 2) and TCF7L2 (transcription factor 7-like 2)), cytoskeleton regulation, and cell migration and adhesion.

The abbreviations in Figure 5 are as follows:

In (a), Dihydropyrimidine dehydrogenase (DPYD); carbamoyl-phosphate synthase 1 (CPS1); carbamoyl-phosphate synthetase 2, aspartate transcarbamylase, and dihydroorotase (CAD); uridine monophosphate synthase (UMPS); collapsin response mediator protein 1 (CRMP1); dihydropyrimidinase-like 3(DPYSL3); dihydropyrimidinase-like 2 (DPYSL2); semaphorin 3A (SEMA3A); cyclin-dependent kinase 5 (CDK5) and FYN is a 59-kDa member of the Src family of kinases typically associated with T-cell and neuronal signaling in development and normal cell physiology.

In (b), calmodulin 2(CALM2); calmodulin 1 (CALM1); calmodulin 3 (CALM3); mitogen-activated protein kinase 3 (MAPK3); caspase 3 (CASP3); mitogen-activated protein kinase 1(MAPK1); death-associated protein kinase 1 (DAPK1); CCAAT/enhancer binding protein (CEBPB); netrin 1 (NTN1); Death-associated protein kinase 3 (DAPK3) and unc-5 homolog B (UNC5B).

In (c), thrombospondin 1 (THBS1); inter-alpha-trypsin inhibitor heavy chain family, member 4 (ITIH4); extracellular matrix protein 1 (ECM1); secreted protein, acidic, cysteine-rich (osteonectin) (SPARC); fibronectin 1 (FN1); vascular endothelial growth factor A (VEGFA); tissue inhibitor of metalloproteinase 3 (TIMP3); epidermal growth factor (EGF); tissue inhibitor of metalloproteinase 1 (TIMP1); lectin, galactoside-binding, soluble, 3 binding protein (LGALS3BP); multimerin 1 (MMRN1);

In (d), matrix metallopeptidase 2 (MMP2); ephrin receptor B6 (EPHB6); ephrin-A1 (EFNA1); ephrin-B1 (EFNB1); ephrin receptor B4 (EPHB4); ephrin receptor B3 (EPHB3); ephrin receptor B2 (EPHB2); ephrin-A2 (EFNA2); ephrin-A3 (EFNA3); ephrin-A4 (EFNA4) and ephrin-A5 (EFNA5);

In (e), pre-B-cell leukemia homeobox 2 (PBX2); pre-B-cell leukemia homeobox 4 (PBX4); Homeobox B5 (HOXB5); meis homeobox 2 (MEIS2); homeobox B7 (HOXB7); meis homeobox 1 (MEIS1); pbx/knotted 1 homeobox 1 (PKNOX1); pre-B-cell leukemia homeobox 1 (PBX1); pre-B-cell leukemia homeobox 3 (PBX3); death-associated protein kinase 1 (DAPK1) and fibroblast growth factor 2 (FGF2).

In (f), transcription factor 7 (TCF7); transcription factor 7-like 1 (TCF7L1); transcription factor 7-like 2 (TCF7L2); lymphoid enhancer-binding factor 1 (LEF1); c-terminal binding protein 1 (CTBP1); transducin-like enhancer of split 2 (TLE2); catenin (cadherin-associated protein), beta 1 (CTNNB1); notch homolog 1 (NOTCH1); histone deacetylase 1 (HDAC1); recombination signal binding protein for immunoglobulin kappa J region (RBPJ) and hairy and enhancer of split 1 (HES1).

Some reported genes (including LGALS3BP, DPYSL2, EPHB4, DAPK1, EFNA1, MAPK1, FGFR1 (fibroblast growth factor receptor 1), CRMP1, and SPARC) belong to cell adhesion and migration regulators. Moreover, among these genes, some are also related to cytoskeleton regulation (DPYSL2, MAPK1 and CRMP1). However, the roles of these genes in tumor-subtype classifiers are not obvious and still need to be experimentally validated.

In addition to the top 30 genes, many of the remaining reported genes were experimentally proved to be associated with the tumorigenesis process [39]. For example, LTA (lymphotoxin alpha) has been proved to be highly related with non-Hodgkin lymphoma; for ANXA1 (annexin A1), the loss of function or expression of this gene has been detected in multiple tumors; and the gene product of SPARC has been correlated with metastasis based on changes to cell shape that can promote tumor cell invasion. It is possible that those genes were altered in tumors, but play weaker roles in classifying different tumor subtypes of SRBCT.

## 3. Materials and Methods

### 3.1. Datasets

Twelve gene-expression datasets containing MVs, which were obtained by using cDNA microarrays technologies [40,41,42,43,44,45,46,47], are included in this work. Those cancer gene datasets proved that downstream classification is insensitive with MV imputation [21]. Thus, it is coincident with the motivation of this study. In this paper, data were normalized into [−0.9, 0.9] with min–max normalization (x′ = −0.9 + (x − min)/(max − min) × 1.8). Specifications of the 12 datasets are given in Table 7.

The alizadeh dataset [40] mainly includes three classes: diffuse large B-cell lymphoma (DLBCL), follicular lymphoma (FL), and chronic lymphocytic leukaemia (CLL). DLBCL contains two subtypes: “germinal centre B-like DLBCL” (DLBCL1) and “activated B-like DLBCL” (DLBCL2).

The bredel dataset [41] has three classes: oligodendroglia-enriched tumor (OG), glioblastoma (GBM), and word health organization grades 1–3 astrocytic tumors (A).

Chen [42] has two classes: hepatocellular carcinoma (HCC), and nontumor liver (liver).

Garber [43] has four classes: small cell lung cancer (SCLC), adenocarcinoma (AC), large cell lung cancers (LCLC), and squamous cell carcinomas (SCC).

Lapointe [44] mainly includes two classes: primary prostate tumors (PT), which include three subtypes (PT1, PT2, PT3), and normal prostate specimens (normal).

Liang [45] has three classes: glioblastoma multiforme (GBM), oligodendroglioma (ODG), and normal brain samples (normal).

Risinger [46] has four classes: papillary serous (PS), clear cell (CC), type I molecular alteration (E), and normal endometrium (N).

Dataset tomlins [47] includes stromal from individuals with no history of prostate disease (STROMA_NOR), stromal nodules of benign prostatic hyperplasia (BPH) (STROMA_BPH), stroma adjacent to prostate cancer foci (STROMA_PCA), epithelium (EPI) from individuals with no history of prostate disease (EPI_NOR), epithelium from nodules of BPH (EPI_BPH), epithelium from individuals with prostate cancer (EPI_ADJPCA), atrophic epithelium (EPI_ATR) including proliferative inflammatory atrophy (PIA) (EPI_ATR_PIA), prostatic intraepithelial neoplasia (PIN), localized metastatic prostate cancer (MET) and hormone-naïve metastatic prostate cancer (MET_HN) or hormone-refractory metastatic prostate cancer (MET_HR).

### 3.2. Design and Analytical Flowchart

**Figure 6:**
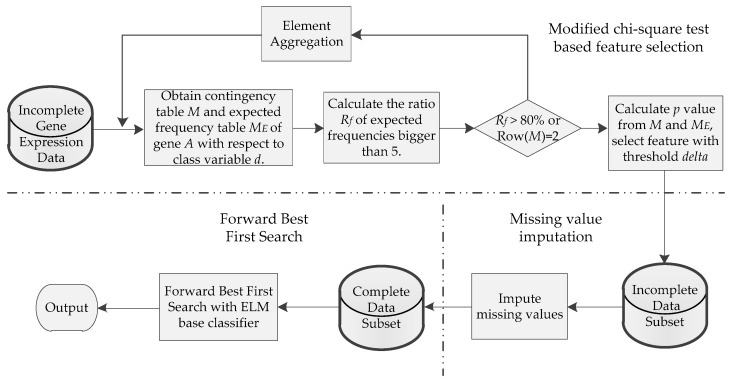
Flowchart of the proposed method. The dotted line divide the framework into three main steps.

The flowchart of our proposed method (first best based on modified chi-square test feature selection: FB-MCFS) is shown in Figure 6. It mainly includes three steps: modified chi-square test-based feature selection (MCFS), missing value imputation and the forward best-first search procedure. In MCFS, a modified chi-square test procedure is introduced to evaluate the importance degree (*p* value) of each gene of the original incomplete expression dataset. Moreover, to meet the small sample size challenge of cancer gene-expression data, a heuristic recursive element aggregation process was proposed to make the chi-square approximation more accurate (need activation conditions). Genes were then selected with a given parameter to construct an incomplete data subset. After that, missing-value imputation was conducted on the selected incomplete data subset to generate a complete data subset. Finally, forward best-first search strategy was utilized to identify the best top percentage of genes on the selected feature subset (complete) with extreme learning machine as the base classifier.

### 3.3. Extreme Learning Machine (ELM)

ELM is a new emerged technique for single-layer forward networks (SLFN) in the past decades; it features much faster training speed and better generalization performance over traditional learning techniques. It is also a special type of neural network. ELM can analytically determine output weights by randomly selecting weights and biases for hidden nodes by using the least-square method without time-consuming learning iterations.

For an arbitrary training set consisting of *N* samples (*x_i_*, *y_i_*) with $x_{i}\in R^{d_{1}}$ and $y_{i}\in R^{d_{2}}$, the output of an SLFN with M hidden neurons is:

(1) $$\begin{array}{cc} y_{i}=\sum_{j=1}^{M}\beta_{j}g(\omega_{j},b_{j},x_{i}),\; & i=1,2,\ldots ,N \end{array}$$

where *g*(.) is the hidden activation function; and $\beta_{j}\in R^{d_{2}}$, $\omega_{j}\in R^{d_{1}}$, and $b_{j}\in R$ are the learning parameters of the *j*th hidden node, respectively.

For all *N* samples, a compact form of system (1) can be written as:

(2) Hβ=Y

where *H* is the output matrix, and $H_{ij}=g(\omega_{j},b_{j},x_{i})$, $\beta =(\beta_{1},\beta_{2},\ldots ,\beta_{M})$ and $Y=(y_{1},y_{2},\ldots ,y_{N})$.

Let *T* = (*t_1_*, *t_2_*… *t_N_*) be the target output matrix. To minimize network cost function ||*Y*-*T*||, ELM claims that, with randomly initialized input weights and biases for the SLFN, System (1) becomes a linear model and output weights can be analytically determined by finding a least-square solution of linear System (1) as:

(3) $$\beta =H^{†}T$$

where $H^{†}$ is the Moore–Penrose generalized inverse [48] of hidden-layer output matrix *H*.

More details about the theoretical proofs of the ELM are in the original paper [49]. The universal approximation property of the ELM is also presented in Reference [50] to support the algorithm. Because weights and biases remain unchanged during the training phase, some parameters may be non-optimal. Some samples near the classification boundary may be misclassified by ELM. So, to reduce the number of such misclassified samples, a voting-based ELM (V-ELM) is proposed by Cao [51]. In this work, we applied V-ELM as the base learning algorithm.

### 3.4. MCFS for Incomplete Data

Given an incomplete dataset *D*, we used ‘?’ to denote the MVs. Let *A* be a feature of *D* that has *m* values (except for ‘?’), and *d* is the class variable with *l* values (except for ‘?’). For each pair of (*a_i_*, *d_j_*), *a_i_* is a value of feature *A* and *d_j_* is a value of *d*. The occurrence count of (*a_i_*, *d_j_*) (denoted by *f_ij_*) is increased by fractions of numbers of occurrences (*?*, *d_j_*), (*a_i_*, *?*) and (*?*, *?*) for features (*A*, *d*) [29].

(4) $$f_{ij}^{\prime }\leftarrow f_{ij}+f_{i(l+1)}\times \frac{col_{j}}{N}+f_{(m+1)j}\times \frac{row_{i}}{N}+f_{\left(m+1)(l+1\right)}\times \frac{f_{ij}}{N}$$

where $f_{ij},\;f_{(m+1)j},\;f_{i(l+1)},\;f_{\left(m+1)(l+1\right)}$ are the observation frequencies of (*a_i_*, *d_j_*), (*?*, *d_j_*), (*a_i_*, *?*) and (*?*, *?*); $row_{i}=\sum_{j=1}^{l}f_{ij},\;col_{j}=\sum_{i=1}^{m}f_{ij},N=\sum_{i=1}^{m}\sum_{j=1}^{l}f_{ij}$.

Construct a contingency table (Table 8) *M* (*m* × *l*) based on the *f_ij_* obtained from system (1).

Then, expected frequency *E_ij_* can be calculated:

(5) $$E_{ij}=r_{i}.\;c_{j}/N$$

where $r_{i}=\sum_{j=1}^{l}f_{ij}$, $c_{j}=\sum_{i=1}^{m}f_{ij}$ are the corresponding row summation and column summation of *M*.

The chi-square statistic value of (*A*, *d*) can be obtained by

(6) $$\chi_{\left(A,d\right)}^{2}=\sum_{i=1}^{m}\sum_{j=1}^{l}\frac{{\left(E_{ij}-f_{ij}\right)}^{2}}{E_{ij}}$$

The *p*-value can be computed from $\chi_{\left(A,d\right)}^{2}$ and the freedom degree (*m* − 1) × (*l* − 1) [52]. A larger *p*-value means a smaller relevance degree of *A* with respect to *d*. In the FS scenario, a given significance level value α is applied to select features with *p*-value smaller than it, and the features can also be sorted by *p*-value (ascending order with *p*-value means the descending order with relevance degree).

**Example** **1.** *We present an example table with missing values to illustrate the chi-square-based feature-evaluation algorithm for incomplete data (Table 9). u_1_, u_2_, u_3_, u_4_, u_5,_ and u_6_ are records. a_1_, a_2,_ and a_3_ are features, and d is the class variable.*

We consider constructing the contingency table of *a_1_* with respect to *d*.

(1) Count occurrence frequencies and construct the frequency table as Table 10:

(2) Calculate the following summations of the frequency table:

Summation of rows: $r_{1}=\sum_{j=1}^{2}f_{1j}=1+1=2$, *r_2_* = 2, *r_3_* = 2;

Summation of columns: *c_1_* = 4, *c_2_* = 2;

$N=\sum_{i=1}^{3}\sum_{j=1}^{2}f_{ij}=6$ (*f_ij_* denote the element of the *i*_th_ row and the *j_th_* column);

(3) Update element *f_ij_* (i∈[1,2,3], j∈[1,2]): $f_{ij}^{\prime }\leftarrow f_{ij}+f_{i(l+1)}\times \frac{col_{j}}{N}+f_{(m+1)j}\times \frac{row_{i}}{N}+f_{\left(m+1)(l+1\right)}\times \frac{f_{ij}}{N}$.

We have: $f_{11}^{\prime }=f_{11}+f_{13}\times \frac{c_{1}}{6}+f_{41}\times \frac{r_{1}}{6}+f_{43}\times \frac{f_{11}}{6}=1+0+1\times \frac{2}{6}+0=\frac{4}{3}$.

Similarly, we have $f_{12}^{\prime }=\frac{4}{3}$, $f_{21}^{\prime }=\frac{7}{3}$, $f_{22}^{\prime }=\frac{1}{3}$, $f_{31}^{\prime }=\frac{4}{3}$ and $f_{32}^{\prime }=\frac{4}{3}$.

(4) Construct contingency table *M* ($M_{ij}=f_{ij}{}^{\prime }$) as Table 11:

### 3.5. Recursive-Element Aggregation

Gene-expression data often have a small number of samples; this presents a challenge to the chi-square test. A chi-square test needs 80% of elements of the expected frequencies bigger than 5, and this approximation breaks down if expected frequencies are too low [25]. To meet this characteristic of gene-expression data, we propose a recursive-element aggregation algorithm to make the approximation more accurate.

For a contingency table *M* as in Table 2, let *f_ij_* be the element of the *i_th_* row and *j_th_* column of *M*. Let *M_E_* be the expected frequency table which is composed of the expected frequencies corresponding to the elements of *M*. Suppose *E_ij_* is the smallest expected frequency corresponding to *f_ij_*. 

Our purpose is to test the dependence between a gene and the class variable. For a contingency table that did not satisfy the condition of the chi-square test, we merged the row with its adjacent row to aggregate elements. Algorithms 1 gives the process of recursive-element aggregation.
**Algorithm 1.** Recursive element aggregation for small sample size.**Input**: Contingency table *M*. **Output**: Optimized contingency table *M’*. 1 Obtain corresponding expected frequency table *M_E_*; 2 Calculate ratio (*R_f_*) of expected frequencies bigger than 5 in *M_E_*; 3 **While** ((*R_f_* < 80%) && (rows of *M* > 2)) { 4 Find the smallest element of *M_E_* (eg: *f_ij_*); 5 Merge the *i_th_* row of *M* with its adjacent row which has more elements smaller than 5; 6 Update *M*, *M_E_*; } 7 *M’*←*M*. 8 **return**
*M’*.

**Figure 7:**
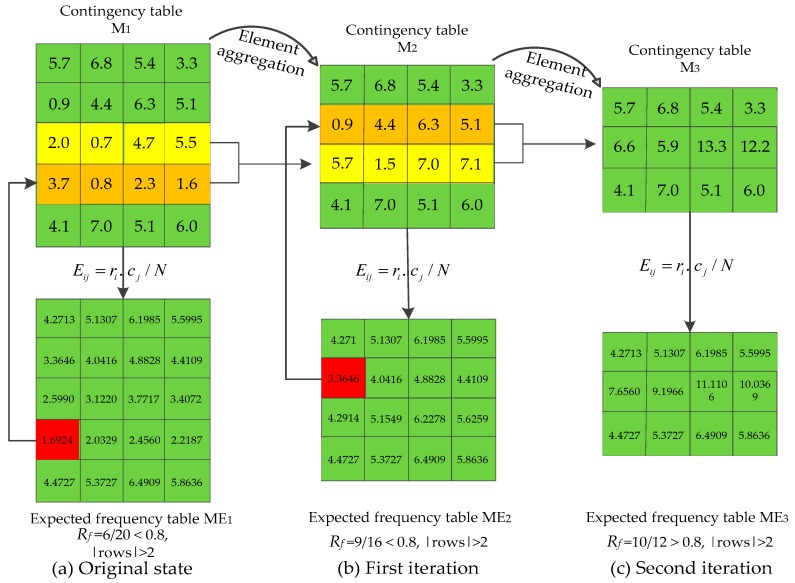
Case for recursive-element aggregation process.

Figure 7 gives an example to describe recursive-element aggregation. In Figure 7a, expected frequency table *ME*_1_ can be obtained from *M*_1_ according to Equation (5). After that, ratio (*R_f_*) of expected frequencies bigger than 5 in *ME*_1_ is calculated. Here, *R_f_* < 0.8, so element aggregation was activated. The smallest element (marked in red background) in *ME*_1_ was firstly found, and then the corresponding row in contingency table *M*_1_ (marked in orange background) was merged with its adjacent row (marked in yellow background), which has more elements smaller than 5. By doing this, we have contingency table *M*_2_. The above processes were iteratively conducted until *R_f_* > 0.8, or the number of rows of the contingency table equalled to two. In Figure 7c, *R_f_* > 0.8, so the element-aggregation process was terminated, and contingency table *M*_3_ was applied to calculate the chi-square statistic value according to Equation (6).

Element aggregation is a heuristic process. After element aggregation, an optimized contingency table can be used to calculate the *p*-value based on Equation (6). We used the MCFS to get the *p*-value of feature *A* with respect to *d*, and then features were sorted in ascending order with *p*-value (the descending order with relevance degree). In this study, a threshold was given to determine whether a feature was removed or selected.

### 3.6. Forward Best First Search on the MCFS Feature Subset 

In this work, incomplete genes with a small relevance degree were removed by MCFS. The selected features may still have redundant genes, so we applied Best First Search (BFS) to search the best top R% features on selected feature subsets. BFS is a standard search technique [53] that can be divided into two categories: Forward BFS (FBFS) and Backward BFS (BBFS). In recent years, the BFS technique has been successfully applied in bioinformatics [54,55]. FBFS is more efficient than BBFS in general; thus, we designed an FBFS strategy on the MCFS feature subset in this subsection.

**Figure 8:**
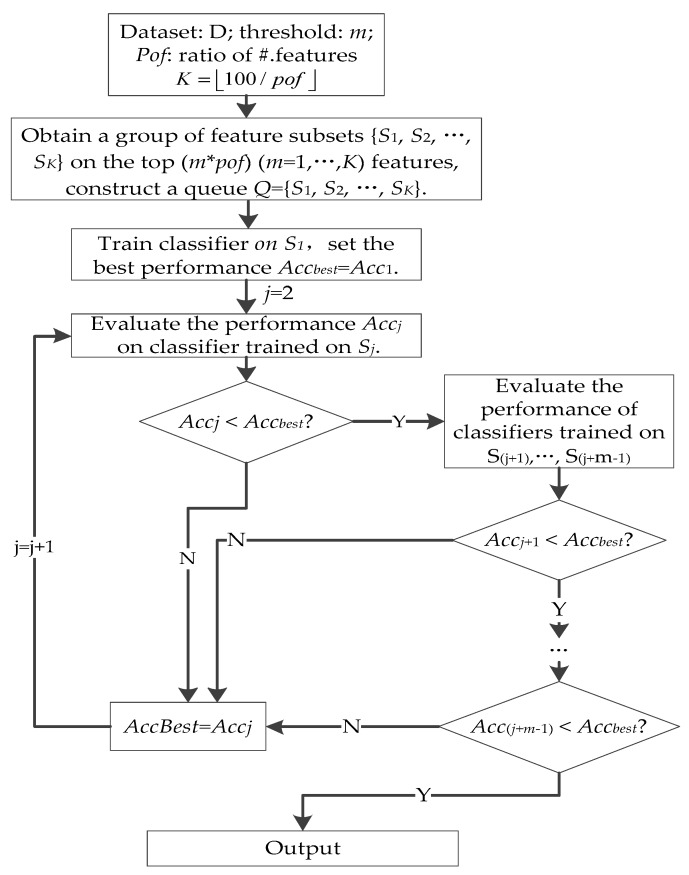
Flowchart of Forward Best-First Search (FBFS) strategy on modified chi-square test-based feature selection (MCFS) subsets.

Genes were ranked according to *p*-value from smallest to biggest (relevance degree from biggest to smallest). FBFS begins with an empty set, and genes in the MCFS feature subset were selected from top-*R*% to 100% with the increment of *R*%. For each round, we first imputed the MVs, then we conducted V-ELM [51] on the data subsets and evaluated V-ELM performance. If performance did not begin to increase, the following *k* rounds were still evaluated. If the current best performance could not be improved, then the FBFS was terminated. For a given ratio of #.features (*pof*), a group of feature subsets {*S*_1_, *S*_2_ … *S_K_*} (*K* = 100/*pof*) could be obtained. Figure 8 gives the flowchart of the FBFS method on the MCFS feature subset.

### 3.7. Comparison Settings

Tenfold cross-validation was applied in this section. V-ELM was applied as the base learning algorithm with a sigmoid activation function. We set 30 as the independent training number of ELM based on a study [51]. Thirty experiment trails were conducted for each hidden node, and the average results are reported. As an FS method, performance of the MCFS was firstly compared with several FS algorithms (neighborhood component feature selection: NCA [56,57], unsupervised feature filtering: UFF [36], and ReliefF [58]) to validate feasibility in terms of FS. To enrich the comparison, PCA [59], which belongs to feature extraction, was also applied in this paper. NCA is a filter-based algorithm, and features with scores larger than 0.000001 were selected in this work. For PCA, the cumulative summation with a top-*k* biggest eigenvalue bigger than 0.999 was used to transform the original data into a new feature space. UFF is an unsupervised FS algorithm. For a feature *i*, UFF scores it using a leave-one-out calculation of (singular value decomposition) SVD entropy to illustrate the possible impact on the FS algorithm with different beforehand MV imputation methods. For ReliefF, we set *k* = 4, and features with weights bigger than 0.05 were selected in this work. For NCA, PCA, UFF, and ReliefF, the imputation method was first applied to construct a complete matrix for the following FS. For our method, MV imputation was also conducted on feature subsets selected by MCFS. Considering that, through removing the most irrelevant genes, features selected by MCFS might still have some redundant genes with the others. This work proposes a forward best-first search-based framework for the MCFS feature subsets.

In this study, Accuracy, Balanced accuracy [60], Recall, Specificity, Precision, F1, and G-mean [61] were used as the main evaluation indicators.

(7) Accuracy=(TP+TN)/(TP+TN+FP+FN)

(8) Recall=TN/(TN+TP)

(9) Specificity=TN/(FP+TN)

(10) Precision=TP/(TP+FP)

(11) Balanced accuracy=(Precision+Recall)/2

(12) F1=2*recall*precision/(recall+precision)

(13) G−mean=Recall+Specificity

where *TP* stands for true-positive samples; *TN* stands for true-negative samples; *FP* stands for false-positive samples; *FN* stands for false-negative samples. The Area Under ROC Curve (AUC) was also reported in this study.

## 4. Discussion

Microarray technology enables researchers to investigate and address issues that were once thought to be non-traceable. Gene-expression data are important data obtained from microarray experiments. However, gene-expression data often encounter missing data and high-dimensional problems. Thus, MV recovery and FS have been two basic types of study of gene-expression data in bioinformatics over the past two decades. Recent studies show that, for real data, MV imputation has minor impact on downstream classification tasks, but MV imputation is based on the MAR assumption; however, the impact of MV imputation on subsequent FS is seldom considered.

We have investigated the impact of different MV imputation methods on subsequent FS in terms of classification. Three imputation methods and several FS algorithms were evaluated in this study. We have found that, for classification tasks, MV imputation has greater influence on subsequent FS in terms of classification performance. Through directly conducting FS on incomplete data, and then filling the missing data on the selected dataset, our approach can avoid the potential influence of beforehand MV imputation on subsequent FS in classification performance. With a proper threshold, our FS algorithm can remove most irrelevant genes, which could make downstream analysis (classification) more efficient.

As a filtering FS algorithm, the dataset selected by our approach may not be optimal. We believe that the real relevance degree of some genes cannot be accurately measured because of missing data in the original datasets. Thus, we suggest to select a few more genes by setting a relatively larger threshold (smaller relevance degree) to avoid possible discarding of potential genes with a relatively higher importance degree. After that, we utilized wrapper methodology and forward best-first search strategy to identify the most informative genes on the selected data subset. It should be emphasized that our criterion is applicable to both complete and incomplete datasets.

Our algorithm proposes to use a heuristic recursive-element aggregation procedure to increase the accuracy of the downstream chi-square approximation. When the size of the gene-expression data sample is very small, the element aggregation procedure is likely to be prematurely terminated (rows of contingency table equaling to two). At this instance, our method cannot reach a satisfying result in evaluating *p*-value.

Biological inference on SRBCT was also conducted to study whether our criterion is biologically meaningful. Many genes selected by our method agreed with the genes found in an (Artificial Neural Network) ANN-based technique and a UFF criterion. Moreover, some of the genes were found in several validated PPI networks, which indicates the importance of the identified biological process in classification tasks. Our criterion also suggests potential new features. However, the roles of these potential genes in tumor subtypes are not obvious, and still need to be experimentally validated.

## Figures and Tables

**Table 1:** Number of selected genes with respect to different thresholds.

Dataset	0.01	0.005	0.001	0.0005	0.0001	0.00001
alizadeh-v1	215	151	72	48	23	7
alizadeh-v2	1811	1534	1077	924	589	306
alizadeh-v3	1771	1535	1049	891	560	262
bredel	2904	2324	809	551	215	63
chen	5759	5007	3714	3276	2499	1700
garber	2125	1494	705	512	231	75
lapointe-v1	3834	2875	1238	875	349	85
lapointe-v2	7615	6113	3696	3012	1895	957
liang	2349	1302	717	623	17	3
risinger	681	419	114	61	16	1
tomlins-v1	4699	3874	2460	1976	1284	570
tomlins-v2	2650	2874	1678	1320	745	335

**Table 2:** Accuracies of a voting-based extreme-learning machine (V-ELM) under different threshold.

Dataset	0.01	0.005	0.001	0.0005	0.0001
alizadeh-v1	1.0000	1.0000	1.0000	1.0000	1.0000
alizadeh-v2	1.0000	1.0000	1.0000	1.0000	1.0000
alizadeh-v3	0.9379	0.9390	0.9445	0.9455	0.9491
bredel	0.8653	0.8650	0.8690	0.8739	0.8782
chen	0.9578	0.9606	0.9638	0.9751	0.9751
garber	0.9009	0.9014	0.9024	0.9060	0.9035
lapointe-v1	0.8732	0.8767	0.8827	0.8887	0.9211
lapointe-v2	0.8679	0.8685	0.8670	0.8699	0.8709
liang	0.9751	0.9760	0.9822	0.9830	0.9575
risinger	0.8577	0.8627	0.8672	0.8852	0.8242
tomlins-v1	0.8855	0.8895	0.8960	0.9048	0.9135
tomlins-v2	0.8866	0.8874	0.8914	0.8944	0.9105

**Table 3:** Accuracy with beforehand/afterward MCFS under three imputation methods.

Datasets	BPCA		KNN		MEAN	
	MCFS1	MCFS2	MCFS1	MCFS2	MCFS1	MCFS2
alizadeh-v1	**1.0000**	0.9928	**1.0000**	0.9956	**1.0000**	0.9944
alizadeh-v2	0.9967	**0.9981**	**1.0000**	1.0000	**0.9971**	0.9949
alizadeh-v3	**0.9565**	0.9461	**0.9503**	0.9486	**0.9449**	0.9432
bredel	**0.8638**	0.8579	**0.8706**	0.8481	**0.8719**	0.8644
chen	**0.9701**	0.9597	**0.9679**	0.9641	**0.9677**	0.9581
garber	**0.9074**	0.9011	0.8889	**0.8986**	0.9054	**0.9071**
lapointe-1	0.8523	**0.8524**	0.8533	**0.8549**	0.8492	**0.8516**
lapointe-2	**0.8621**	0.8470	**0.8583**	0.8565	0.8506	**0.8511**
liang	**0.9923**	0.9863	0.9860	**0.9863**	0.9820	**0.9856**
risinger	0.8643	**0.8659**	0.8575	**0.8589**	0.8656	**0.8663**
tomlins-v1	**0.8879**	0.8809	**0.8892**	0.8792	**0.8965**	0.8847
tomlins-v2	**0.8850**	0.8678	**0.8943**	0.8637	**0.8839**	0.8776
Average	**0.9199**	0.9130	**0.9180**	0.9129	**0.9179**	0.9149

Bold: best performance.

**Table 4:** Performance comparison of FS algorithms under the three MV imputation methods.

Datasets	NCA			PCA			UFF			ReliefF			MCFS		
	BPCA	KNN	MEAN	BPCA	KNN	MEAN	BPCA	KNN	MEAN	BPCA	KNN	MEAN	BPCA	KNN	MEAN
ali1	**0.9400**	**0.9870**	**0.9820**	**0.8550**	**0.8390**	**0.8270**	**0.8390**	**0.7770**	**0.7840**	1.0000	1.0000	1.0000	1.0000	1.0000	1.0000
ali2	1.0000	1.0000	1.0000	0.9748	0.9933	0.9818	0.9023	0.9023	0.8860	1.0000	1.0000	1.0000	0.9967	1.0000	0.9971
ali3	**0.9625**	**0.9892**	**0.9672**	0.8893	0.8952	0.8882	0.8138	0.8209	0.8156	0.9722	0.9746	0.9686	0.9565	0.9503	0.9449
bredel	**0.9369**	**0.7689**	**0.9601**	0.7840	0.7994	0.7931	/	/	/	**0.8605**	**0.8432**	**0.8303**	0.8638	0.8706	0.8719
chen	0.9833	0.9925	0.9946	0.9379	0.9316	0.9374	0.9448	0.9422	0.9475	0.9877	0.9865	0.9835	0.9701	0.9679	0.9677
garber	0.9327	0.9496	0.9242	0.7837	0.7985	0.7860	**0.7944**	**0.7680**	**0.7734**	0.8896	0.8965	0.9036	0.9074	0.8889	0.9054
lap1	**0.9416**	**0.9722**	**0.9466**	0.7176	0.7352	0.7202	0.7048	0.7052	0.7110	**0.8464**	**0.8570**	**0.8833**	0.8523	0.8533	0.8492
lap2	0.9353	0.9431	0.9401	0.7270	0.7324	0.7135	0.7169	0.7355	0.7285	0.9011	0.8948	0.9060	0.8621	0.8583	0.8506
liang	1.0000	1.0000	1.0000	**0.9423**	**0.9553**	**0.9223**	**0.8813**	**0.9097**	**0.9143**	1.0000	1.0000	1.0000	0.9923	0.9860	0.9820
risinger	0.8693	0.8679	0.8829	**0.7067**	**0.6833**	**0.6910**	**0.7102**	**0.7180**	**0.6849**	0.8267	0.8323	0.8427	0.8643	0.8575	0.8656
tom1	**0.9259**	**0.9010**	**0.9172**	**0.7833**	**0.8381**	**0.7956**	**0.2812**	**0.8305**	**0.4093**	**0.9191**	**0.9202**	**0.8984**	0.8879	0.8892	0.8965
tom2	**0.9304**	**0.8937**	**0.9122**	**0.7389**	**0.8144**	**0.7435**	**0.4499**	**0.8168**	**0.4364**	**0.8859**	**0.8997**	**0.8794**	0.8850	0.8943	0.8839

Bold: performance with more than 2% differences under the 3 imputation methods.

**Table 5:** Summary of Friedman *p*-values between FB-MCFS and the other algorithms under three imputation methods.

	BPCA	KNN	MEAN
NCA	1	0.5271	0.5236
PCA	**0.0016**	**0.0044**	**0.0014**
UFF	**1.478 × 10^−4^**	**5.0422 × 10^−4^**	**4.5173 × 10^−4^**
ReliefF	0.0578	0.0578	0.0557
MCFS	**0.0016**	**0.0044**	**0.0041**

Bold: friedman *p*-values smaller than 0.05.

**Table 6:** Top 30 genes selected by MCFS.

MCFS Ranking	Gene Name	Ref [37]’s Ranking	UFF Ranking
1	‘growth arrest-specific 1’	4	33
2	‘selenium binding protein 1’	63	/
3	‘cyclin D1 (PRAD1: parathyroid adenomatosis 1)’	3	11
4	‘olfactomedin related ER (endoplasmic reticulum) localized protein’	19	23
5	‘recoverin’	29	/
6	‘thioredoxin’	/	/
7	‘quinone oxidoreductase homolog’	61	/
8	‘glycogen synthase 1 (muscle)’	/	/
9	‘amyloid precursor-like protein 1’	32	/
10	‘ESTs (EST: expressed sequence tag), Moderately similar to skeletal muscle LIM-protein (named for ‘LIN11, ISL1, and MEC3,’) FHL3 (FHL: four-and-a-half lim domains 3) (H.sapiens)’	/	/
11	‘type II integral membrane protein’	/	/
12	‘GLI (glioma-associated oncogene homolog)-Kruppel family member GLI3 (Greig cephalopolysyndactyly syndrome)’	/	/
13	‘transducin-like enhancer of split 2, homolog of Drosophila E(sp1)’	35	/
14	‘interferon-inducible’	44	78
15	‘calponin 3, acidic’	5	83
16	‘Fc (fragment, crystallizable) fragment of IgG (immunoglobulin G), receptor, transporter, alpha’	6	50
17	‘protein tyrosine phosphatase, non-receptor type 12’	/	/
18	‘cold shock domain protein A’	/	/
19	‘antigen identified by monoclonal antibodies 12E7, F21 and O13’	73	44
20	‘lectin, galactoside-binding, soluble, 3 binding protein (galectin 6 binding protein)’	20	/
21	‘Cbp/p300-interacting transactivator, with Glu/Asp-rich carboxy-terminal domain, 2’	/	/
22	‘dihydropyrimidinase-like 2’	60	/
23	‘suppression of tumorigenicity 5’	/	/
24	‘complement component 1 inhibitor (angioedema, hereditary)’	51	48
25	‘caveolin 1, caveolae protein, 22kD’	18	18
26	‘homeo box B7’	/	/
27	‘guanine nucleotide exchange factor; 115-kD; mouse Lsc homolog’	/	/
28	‘EphB4 (ephrin type-B receptor 4)’	/	/
29	‘death-associated protein kinase 1’	82	/
30	‘insulin-like growth factor 2 (somatomedin A)’	1	2

**Table 7:** Specification of cancer gene-expression data.

Dataset	Array Type	Tissue	Dimensionality	Samples per Class	Classes
alizadeh-v1	Double Channel	Blood	4026	21,21	DLBCL1, DLBCL2
alizadeh-v2	Double Channel	Blood	4026	42, 9, 11	DLBCL, FL, CLL
alizadeh-v3	Double Channel	Blood	4026	21, 21, 9, 11	DLBCL1, DLBCL2, FL, CLL
bredel	Double Channel	Brain	41472	31, 14, 5	GBM, OG, A
chen	Double Channel	Liver	24192	104, 75	HCC, liver
garber	Double Channel	Lung	24192	17, 40, 4, 5	SCC, AC, LCLC, SCLC
lapointe-v1	Double Channel	Prostate	42640	11, 39, 19	PT1, PT2, PT3
lapointe-v2	Double Channel	Prostate	42640	11, 39, 19, 41	PT1, PT2, PT3, Normal
liang	Double Channel	Brain	24192	28, 6, 3	GBM, ODG, Normal
risinger	Double Channel	Endometrium	8872	13, 3, 19, 7	PS, CC, E, N
tomlins-v1	Double Channel	Prostate	20000	27, 20, 32, 13, 12	EPI, MET, PCA, PIN, STROMA
tomlins-v2	Double Channel	Prostate	20000	27, 20, 32, 13	EPI, MET, PCA, PIN

**Table 8:** Contingency table *M*.

***A***	***d***		
	*d_1_*	…	*d_l_*
*a_1_*	*f_11_*	…	*f_1l_*
…	…	…	…
*a_m_*	*f_m1_*	…	*f_ml_*

**Table 9:** Example table with missing values.

Sample	Wind (*a1*)	Humidity (*a2*)	Temperature (*a3*)	Trip (*d*)
u1	low	low	high	yes
u2	medium	medium	medium	yes
u3	high	high	?	yes
u4	low	medium	high	no
u5	?	?	high	no
u6	medium	high	low	yes
u7	?	low	low	yes
u8	high	high	high	no

**Table 10:** Frequency table of *a1* with respect to *d*.

*a_1_*(wind)	*d*(trip)		
	Yes	No	?
low	1	1	0
medium	2	0	0
high	1	1	0
?	1	1	0

**Table 11:** Contingence table M.

*a_1_*	*d* (trip)	
	yes	no
low	4/3	4/3
medium	7/3	1/3
high	4/3	4/3

## Supplementary Materials

Supplementary materials can be found at http://www.mdpi.com/1422-0067/19/11/3398/s1.
